# Anticonvulsant and neurotoxicity profile of the rhizome of *Smilax china* Linn. in mice

**DOI:** 10.4103/0253-7613.75662

**Published:** 2011-02

**Authors:** A. Vijayalakshmi, V. Ravichandiran, J. Anbu, Malarkodi Velraj, S. Jayakumari

**Affiliations:** Department of Pharmacognosy, School of Pharmaceutical Sciences, Vels University, Chennai - 117, Tamil Nadu, India

**Keywords:** Epilepsy, China root, Smilax china, neurotoxicity

## Abstract

**Objective:**

To study the anticonvulsant activity and neurotoxicity of ethanolic extract and ethyl acetate fraction of the rhizome of Smilax china (EESC and EAF, respectively) in mice.

**Materials and Methods:**

The anticonvulsant activities of EESC and EAF were studied against maximal electroshock (MES) and pentylenetetrazole (PTZ)-induced seizures in mice and neurotoxicity was determined using rotarod test.

**Results:**

The duration of hindleg extension in MES test was reduced significantly (*P* < 0.001) by EESC at a dose level of 400 mg/kg and EAF at both higher dose levels (200 and 400 mg/kg). In PTZ model, the seizure latency was prolonged by all the test groups.

**Conclusion:**

The EESC and EAF may help to control petit mal and grand mal seizures.

## Introduction

Epilepsy has been a serious disorder that accounts for about 1% of the world’s burden of diseases. A number of synthetic antiepileptic drugs are available. However, their effectiveness does not hold true with the entire range of population. Further, a large number of drug interactions and their side effects make it more difficult to attain easy control on seizures. On the other hand, herbal medicines are widely used due to their applicability and efficacy coupled with least side effects, which in turn has accelerated the scientific research regarding the antiepileptic activity. There is still a need for broadly acting anticonvulsant drugs possessing multiple mechanisms of action with decreased adverse effect, preferably originating from natural products, despite the beneficial effect of currently available drugs.

The rhizomes of *Smilax china* Linn. (Liliaceae),[1] mentioned in Ayurveda, Siddha and Unani systems of medicine, have been used to treat epilepsy, insanity, syphilis, colic, skin diseases, chronic nervous diseases, neuralgia, rheumatism and gout.[2–4] In view of the need for safe and effective herbal antiepileptics, the present study was undertaken to evaluate the anticonvulsant effect of the ethanolic extract of the rhizome of S. *china* (EESC) and its bioactive potent ethyl acetate fraction (EAF) against the seizures induced by maximum electroshock (MES) and pentylenetetrazole (PTZ) in mice.

## Materials and Methods

### 

#### Plant material

The dried plant specimen for the proposed study was purchased from a commercial source, at Chennai, Tamil Nadu. It was identified and authenticated by Dr. P. Jayaraman, Director, Plant Anatomy Research Center, (PARC), Chennai, where the voucher specimen has been deposited (no. 168) in the Pharmacognosy herbarium, Vels University, India.

#### Drugs

Pentylenetetrazole (Sigma, St. Louis, USA), Diazepam (DZP Calmpose inj. Ranbaxy, New Delhi, India) and phenytoin (Ranbaxy, New Delhi, India) were used in this study. DZP injection was diluted to the required volume with distilled water before use. Appropriate vehicle controls were employed in all experiments. The dried extracts and its fraction were suspended in 2% carboxy methyl cellulose (CMC).

#### Extraction

The rhizomes of S. *china* Linn. were dried and coarsely powdered. About 500 g of the powder was extracted with ethanol (70% v/v ethanol) by cold maceration method. The solvent was filtered and distilled off and the final traces of solvent were removed under vacuum. The total extract thus obtained was fractionated successively with the solvents of increasing polarity, viz., hexane, chloroform and ethyl acetate. The solvent was filtered and distilled off. Final traces of solvent were removed under vacuum.[5]

#### Phytochemical screening

Qualitative tests for the presence of plant secondary metabolites, such as carbohydrates, alkaloids, tannins, flavonoids, saponins and glycosides, were carried out on the total extract and its fractions using standard procedures.[5]

#### Acute toxicity

The acute toxicity study of EESC and EAF was performed using up and down procedure at dose levels of 1000 and 2000 mg/kg body weight orally in mice, as per OECD-423 guidelines, in two groups of 10 mice each and were observed for mortality after 24 h.[6] The Institutional Animal Ethics Committee (IAEC) approved the protocol (Registration no. 290 CPCSEA dated 6.10.2008).

#### Potentiation of pentobarbitone-induced loss of righting reflex

Three different experimental groups of six mice each received the EESC, and three different experimental groups (*n* = 6 per group) received the EAF orally at doses of 125, 250 and 500 mg/kg body weight, respectively. The control group received the vehicle. Sodium pentobarbitone (30 mg/kg, i.p.) was injected 1 h after the drug administration and the sleeping time in each animal was determined by recording the time interval between the loss and recovery of the righting reflex. The sleep latency is the duration of time between the administration of pentobarbitone and loss of righting reflex. The righting reflex was considered to be the lost when the animal was placed on its back and failed to regain its normal posture within 10 second. The time of each experimental group was compared with that of the control.[7]

#### Neurotoxicit

The neurotoxicity of the test drug was determined using rotarod test.[8] Rats which were able to remain on the rotating rod at a speed of 10 rpm for 5 min or more were selected and divided into seven groups of six animals in each. Group one received vehicle and served as control and the experimental groups received the EESC and EAF in the same dose. The animals were placed on the rotarod after 1 h of the drug administration. Animals that failed to remain on the rotarod for 3 min were considered as having signal motor incoordination.

#### Assessment of activity

#### MES model

MES model was used to evaluate the anticonvulsant activity of the extract.[9] The seizures were induced by an electroconvulsiometer by delivering electroshock of 60 mA current transauricularly for 0.2 second in mice using corneal electrodes. The test animals received 100, 200 and 400 mg/kg of EESC and EAF orally, 30 min before the application of electroshock. The duration of tonic hind leg extension (THE) and mortality for each animal was observed for 2 and 24 h respectively. Decrease in duration of hind limb extension was considered as a protective action.

#### PTZ model

The animals in each group were pretreated with 100, 200 and 400 mg/kg of EESC and EAF orally. After 30 minutes, PTZ was administered at a dose of 80 mg/kg subcutaneously and observed for the convulsive behavior for 30 min. The parameters measured were onset of myoclonic jerk, latency, clonus, tonic flexion and mortality.[10] The efficacy of the test drug to protect the animals against lethal seizures was measured in terms of extended latency period and decreased percentage of mortality of each group and compared with the respective control group.

#### Statistical analysis

The duration of THE phase of MES convulsions and seizure latency of PTZ-induced seizures was expressed as the arithmetic mean ± SE and was analyzed by one-way analysis of variance (ANOVA), followed by Dunnett’s “t”- test. *P* value less than 0.05 (*P* < 0.05) was the critical criterion for statistical significance.

## Results

### 

#### Phytochemical study

The total ethanolic extract showed positive results for steroids, terpenoids, flavonoids, tannins, glycosides and carbohydrates, the hexane fraction showed positive results for steroids, the chloroform fraction showed positive results for terpenoids and the ethyl acetate fraction showed positive results for flavonoids, tannins, terpenoids and carbohydrates. The total extract and ethyl acetate fraction showed maximum phytoconstituents and hence were selected for the present pharmacological study.

#### Acute toxicity

In the acute toxicity study, both EESC and EAF did not show any mortality up to a dose of 2 g/kg body weight in mice. Even at this high dose, there were no gross behavioral changes.

#### Pentobarbitone-induced loss of righting reflex

Out of the three doses of EESC and EAF administered orally, latency period of sleep was decreased significantly (*P* <0.001) at 250 and 500 mg/kg for both EESC and EAF. The EESC increased pentobarbitone-induced sleeping time significantly at higher doses of 250 and 500 mg/kg. The EAF increased pentobarbitone-induced sleeping time at all tested doses (125, 250 and 500 mg/kg) [Table 1].

**Table 1:** Effect of EESC and EAF on pentobarbitone-induced sleeping time, sleep latency and rotarod test in rats

*Group*	*Treatment*	*Dose*	*Sleep latency (seconds)*	*Sleeping time (min)*	*Rotarod activity (seconds)*
I	Vehicle	1 mL	244.5 ± 3.40	172.33 ± 2.42	258.80 ± 34.00
III	EESC	125 mg/kg	235.83 ± 3.91	181.83 ± 2.78	231.45 ± 23.49
IV	EESC	250 mg/kg	202.17 ± 3.38***	192.33 ± 3.76**	241.70 ± 22.90
V	EESC	500 mg/kg	198.00 ± 2.51***	261.78 ± 1.98***	191.47 ± 16.79
VI	EAF	125 mg/kg	230.17 ± 2.42	197.83 ± 1.06**	226.82 ± 32.45
VII	EAF	2750 mg/kg	177.83 ± 3.91***	375.17 ± 2.66***	237.30 ± 28.87
VIII	EAF	500 mg/kg	124.83 ± 2.76***	364.50 ± 3.78***	206.54 ± 43.80

EESC: Ethanolic extract of *Smilax china* Linn.; EAF: Ethyl acetate fraction; Values are mean ± SEM (n = 6) statistical significance was determined by ANOVA, followed by Dunnett’s “t” test (n =6);

*** *P* < 0.001,

** *P* < 0.01,

^*^P < 0.05 when compared against control ^a^*P* < 0.001, ^b^*P* < 0.01, when compared against standard

#### Neurotoxicity

Administration of EESC and EAF at all doses did not produce any significant effect on motor coordination [Table 1].

#### MES model

Administration of EAF at 100, 200 and 400 mg/kg resulted in hindleg extension for 12.33 ± 0.42, 10.16 ± 0.70 and 8.83 ± 0.30 seconds respectively. The EESC and EAF in all doses did not protect animals from seizures. However, the duration of hind leg extension was reduced more significantly (*P* < 0.001) by 400 mg/kg EESC and 200 - 400 mg/kg EAF. The percentage of mortality protection was highly significant for all tested groups when compared to control [Table 2].

**Table 2:** Effect of EESC and EAF on maximal electroshock induced seizures in mice

*Group*	*Treatment*	*Dose*	*Duration of hind limb extensor phase (seconds)*	*% Mortality*
I	Vehicle	1 mL	16.1 ± 0.75	72.33
II	Phenytoin	20 mg/kg	9.3 ± 0.7***	100**
III	EESC	100 mg/kg	13.66 ± 0.91^a^	94.33*
IV	EESC	200 mg/kg	12.83 ± 0.60**^b^	100**
V	EESC	400 mg/kg	11.30 ± 0.33***	100**
VI	EAF	100 mg/kg	12.33 ± 0.42**^b^	100**
VII	EAF	200 mg/kg	10.16 ± 0.70***^b^	100**
VIII	EAF	400 mg/kg	8.83 ± 0.30***	100**

EESC: Ethanolic extract of *Smilax china* Linn.; EAF: Ethyl acetate fraction; Values are mean ± SEM (n =6). Statistical significance was determined by ANOVA, followed by Dunnett’s “t” test (n=6);

*** *P* < 0.001,

** *P* < 0.01,

* *P* < 0.05 when compared against control

a *P* < 0.001,

b *P* < 0.01,

when compared against standard

#### PTZ model

The seizure latency was prolonged by all the test groups as compared to control. The percentage protection afforded increased at all dose levels of EAF, whereas the duration of tonic flexion and clonus of test groups were significantly (*P* < 0.01) lowered at all dose levels. 100% protection was achieved with 200 and 400 mg/kg EESC and EAF and DZP [Table 3].

**Table 3:** Effect of EESC and EAF on PTZ-induced seizures in mice

*Group*	*Treatment*	*Dose*	*Onset time (seconds)*	*Duration of clonus (seconds)*	*Protection (%)*
I	Vehicle	1 mL	190.33 ± 1.83	8.16 ± 0.40	0
II	Diazepam	2 mg/kg	0	0	100***
III	EESC	100 mg/kg	268.33 ± 13.01^a^	4.83 ± 0.30**	54
IV	EESC	200 mg/kg	298.33 ± 25.48***^a^	14.83 ± 0.30**	100***
V	EESC	400 mg/kg	481.83 ± 2.83***^a^	19.16 ± 0.65**	100***
VI	EAF	100 mg/kg	289.16 ± 7.79***^a^	2.66 ± 0.21**	83**
VII	EAF	200 mg/kg	356 ± 6.42^a^	2.83 ± 0.16**	100***
VIII	EAF	400 mg/kg	495 ± 9.83***^a^	5.5 ± 0.34**	100***

EESC: Ethanolic extract of *Smilax china* Linn.; EAF: Ethyl acetate fraction; Values are mean ± SEM (n = 6). Statistical significance was determined by ANOVA, followed by Dunnett’s “t” test (n=6);

*** *P* < 0.001,

** *P* < 0.01,

^*^P < 0.05 when compared against control

a *P* < 0.001,

when compared against standard

## Discussion

Significant advances are being made in the recent years to treat epilepsy using second-generation drugs.[11] Polypharmacy is often advocated to 30% of all epileptic patients for refractory partial or generalized tonic-clonic seizures.[12] However, none of the new drugs fulfills the ultimate goal of complete control of seizures.[13] Therefore, there is a need for broadly acting anticonvulsant drugs possessing multiple mechanisms of action with lesser adverse effect. Results of the present study indicate that the EESC and EAF at 250 and 500 mg/kg dose levels significantly decrease sleep latency period and increase sleeping time. The exact mechanism by which EESC and EAF potentiate pentobarbitone-induced sleeping time is not known. 5-Hydroxytryptamine (5-HT) mediated stimulation of reticular activating system (RAS) may be implicated in the events that led to prolongation of pentobarbitone-induced sleeping time and reduction of sleep latency.

The present study reveals that the test drugs exhibit a significant dose-dependent protection against electrical and chemical-induced seizure. Gamma-aminobutyric acid (GABA) is a major inhibitory neurotransmitter and the enhancement of GABA neurotransmission has shown to attenuate the convulsion. PTZ may be exerting its convulsive effect by inhibiting the activity of gamma amino butyric acid (GABA) at GABA_A_ receptors, the major inhibitory neurotransmitter which is implicated in epilpsy. DZP, a benzodiazepine, is reported to antagonize PTZ-induced absence seizures by enhancing GABA neurotransmission.[14] Hence, in the present investigation, DZP was employed as a standard drug in PTZ model. It has been indicated that PTZ-induced seizures can be prevented by drugs that reduce T-type Ca^2+^ currents, such as ethosuximide, and also by drugs that enhance GABA_A_ receptor-mediated inhibitory neurotransmission, such as benzodiazepines and phenobarbital.[15] The MES test is considered to be a predictor of likely therapeutic efficacy against generalized tonic-clonic seizures. On the other hand, the PTZ test represents a valid model for human generalized myoclonic and absence seizures.[16] Various chemical constituents of plant origin, such as terpenoids, particularly triterpenoids and flavonoids, are reported to have anticonvulsant property in various animal models of epilepsy like PTZ, MES, electrical kindling, etc.[17,18] Flavonoids are complex chemical molecules found in several medicinal plants. Chrysin, a natural flavonoid identified in *Passiflora coerulea*, is used as a sedative in American continent, and was found to be a ligand for the benzodiazepine receptors.[19] PTZ has been classified as a central benzodiazepine receptor antagonist.[20] Apigenin, a flavonoid, has the ability to selectively bind with high affinity to the central benzodiazepine receptors.[18] These findings suggest that the flavonoid in the EESC and EAF might behave as a partial agonist of benzodiazepine receptors, decreasing the antagonistic effect of PTZ. Thus, our results suggest that flavonoids present in the plant may also be responsible for the effectiveness of the tested drugs against human generalized myoclonic and absence seizures.

Hence, it may be concluded that EESC and EAF inhibit convulsions induced by PTZ and MES models of epilepsy, without affecting motor coordination. The observed anticonvulsant activity may be due to potentiation of GABAergic neurotransmission and/or increase in neuronal seizure threshold by decreased Na^+^channel activity, which may be attributed to the phytoconstituents present in the extracts.
